# Receptive Fields in Primate Retina Are Coordinated to Sample Visual Space More Uniformly

**DOI:** 10.1371/journal.pbio.1000063

**Published:** 2009-04-07

**Authors:** Jeffrey L Gauthier, Greg D Field, Alexander Sher, Martin Greschner, Jonathon Shlens, Alan M Litke, E. J Chichilnisky

**Affiliations:** 1 Salk Institute for Biological Studies, La Jolla, California, United States of America; 2 Santa Cruz Institute for Particle Physics, University of California, Santa Cruz, California, United States of America; 3 University of California, Berkeley, California, United States of America; University of California, Berkeley, United States of America

## Abstract

In the visual system, large ensembles of neurons collectively sample visual space with receptive fields (RFs). A puzzling problem is how neural ensembles provide a uniform, high-resolution visual representation in spite of irregularities in the RFs of individual cells. This problem was approached by simultaneously mapping the RFs of hundreds of primate retinal ganglion cells. As observed in previous studies, RFs exhibited irregular shapes that deviated from standard Gaussian models. Surprisingly, these irregularities were coordinated at a fine spatial scale: RFs interlocked with their neighbors, filling in gaps and avoiding large variations in overlap. RF shapes were coordinated with high spatial precision: the observed uniformity was degraded by angular perturbations as small as 15°, and the observed populations sampled visual space with more than 50% of the theoretical ideal uniformity. These results show that the primate retina encodes light with an exquisitely coordinated array of RF shapes, illustrating a higher degree of functional precision in the neural circuitry than previously appreciated.

## Author Summary

All visual information reaching the brain is transmitted by retinal ganglion cells, each of which is sensitive to a small region of space known as its receptive field. Each of the 20 or so distinct ganglion cell types is thought to transmit a complete visual image to the brain, because the receptive fields of each type form a regular lattice covering visual space. However, within each regular lattice, individual receptive fields have jagged, asymmetric shapes, which could produce “blind spots” and excessive overlap, degrading the visual image. To understand how the visual system overcomes this problem, we used a multielectrode array to record from hundreds of ganglion cells in isolated patches of peripheral primate retina. Surprisingly, we found that irregularly shaped receptive fields fit together like puzzle pieces, with high spatial precision, producing a more homogeneous coverage of visual space than would be possible otherwise. This finding reveals that the representation of visual space by neural ensembles in the retina is functionally coordinated and tuned, presumably by developmental interactions or ongoing visual activity, producing a more precise sensory signal.

## Abstract 3

Receptive fields in primate retina have irregular shapes, but they interlock like puzzle pieces to provide highly uniform coverage of visual space.

## Introduction

In primates, high-resolution visual information is encoded by the magnocellular and parvocellular pathways, which respectively originate in the retina as populations of parasol and midget retinal ganglion cells (RGCs) [1]. These populations are expected to represent the visual scene efficiently and completely. Contrary to this expectation, indirect evidence suggests that the receptive fields (RFs) of individual parasol and midget cells have irregular and inconsistent shapes [2–5], and thus that the visual representation may be patchy, with inhomogeneous gaps and overlap [3]. The problem of uniformly sampling visual space has an intriguing conceptual correlate, and potential solution, in the anatomical literature: in certain ganglion cell types such as primate midget cells, dendritic fields (DFs) are coordinated to uniformly cover the physical surface of the retina [6–8]. However, despite a rough alignment of RF and DF shapes [4,9,10], it is not clear whether these shapes match at a fine spatial scale [3]. Thus the coordination of DFs may or may not produce coordination of RFs. Moreover, the DFs of primate parasol cells overlap substantially, with no obvious signs of coordination of their spatial extent [11]. We set out to test whether and how RGCs in the high-resolution visual pathways of primates are coordinated to transmit a uniformly sampled image to the brain.

## Results

To measure directly how ganglion cell populations sample visual space, large-scale simultaneous recordings were obtained from hundreds of identified neurons in patches of peripheral primate retina [12,13]. Stable recordings over several hours allowed RFs to be mapped at a fine spatial scale. Because hundreds of cells were recorded simultaneously, they could be grouped into clear functional classes defined by physiological properties such as latency, light response polarity, and spike train autocorrelation (see Materials and Methods) [13–15]. These properties, combined with the density of each functional class, were used to identify the distinct classes as on and off parasol and on and off midget cells, morphologically distinct cell types with distinct projection patterns in the brain. Frequently, every cell of a type was recorded in a local region [15], presenting a unique opportunity for the study of collective encoding.

**Figure 1:**
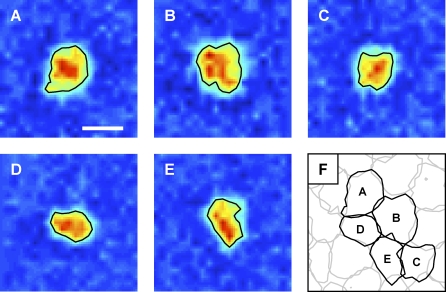
Irregularly Shaped RFs Were Coordinated, Providing More Uniform Sampling of Visual Space (A–E) Receptive fields (RFs) of five simultaneously recorded on parasol cells mapped using white noise exhibited an irregular fine structure that deviates from a simple Gaussian model. Warmer colors indicate greater light sensitivity. For visualization (Figures 1 and 2) and analysis (Figures 3 and 4), each RF was low-pass filtered to suppress measurement noise and summarized by a contour line describing its shape at a single contour level (see Materials and Methods). Surrounds were too weak to be seen in individual cells, but averaging over cells revealed a clear surround in all four cell types (unpublished data). The RFs of off parasol and on and off midget cells had similar irregular shapes (see Figure 2). For visualization in this figure only, pixels were subsampled using linear interpolation by a linear factor of 3. Scale bar indicates 180 μm. (F) The contours of the on parasol cells shown in (A–E).

Parasol and midget cell RF shapes strongly deviated from the theoretical ideal of a smooth surface defined by a difference of Gaussians [16]. In particular, RF shapes exhibited fine structure and irregular outlines, with shapes and sizes varying significantly from cell to cell (Figure 1A–1E), consistent with previous studies [2–5]. The observed irregularity of individual RFs suggested that the collective visual coverage by each cell type might be uneven and irregular, potentially posing a problem for high-resolution vision.

**Figure 2:**
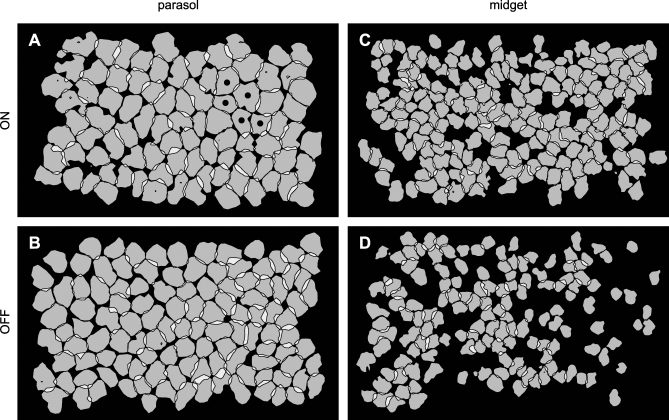
Irregular RF Shapes Were Coordinated in Large Populations of on and off Midget and Parasol Cells The simultaneously recorded RFs of each cell type formed a regularly spaced mosaic, represented here as a collection of contour lines. The contour level was the same for all cells in each mosaic, and was chosen so that neighboring contours, on average, just touched (see Materials and Methods). The width of each panel represents approximately 2.2 mm on the retina. (A) RFs of 88 simultaneously recorded on parasol cells from 9 mm eccentricity (temporal retina). Cells marked with a dot are those shown in Figure 1. (B) RFs of 117 simultaneously recorded off parasol cells from the same preparation as in (A). (C) RFs of 179 simultaneously recorded on midget cells from 8 mm eccentricity (superior retina). (D) RFs of 141 simultaneously recorded off midget cells from 11.5 mm eccentricity (superior-nasal retina).

**Figure 3:**
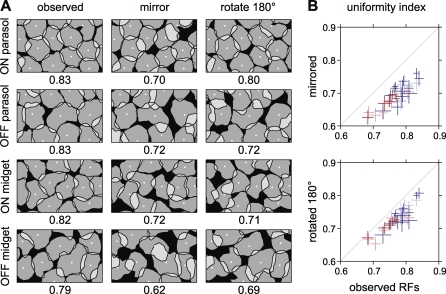
RF Coordination Was Perturbed by Mirroring and Rotation (A) RFs of each cell type are shown at high resolution along with geometric tests of RF coordination. In the observed mosaic (left column), cells appeared to interlock like puzzle pieces. Randomizing the interaction between RF contours by mirroring (center column) or rotating (right column) disrupted visual coverage, demonstrating that fine RF structure is locally coordinated, making visual sampling more uniform (see text). The center point around which RF contours were rotated or mirrored was the center point of an elliptical difference of Gaussians fit. Numbers beneath each panel indicate the UI in this region (see text). The respective horizontal dimensions of the panels for each cell type represent 930, 840, 570, and 330 μm on the retina. (B) Statistical tests demonstrate that RF interlocking was consistent across many preparations. For each population of simultaneously recorded cells of a single type, the UI value is shown for the observed data and RFs that were mirrored or rotated (see text). On parasol data are shown in light blue, off parasol in dark blue, on midget in light red, and off midget in dark red. In every population, the UI decreased when RF contours were mirrored or rotated, demonstrating that fine RF structure is coordinated with neighbors. Each population was composed of 34 to 239 (mean 98) simultaneously recorded cells, for a total of 3,140 cells from 32 populations. Error bars represent the SEM within each population (see Materials and Methods).

**Figure 4:**
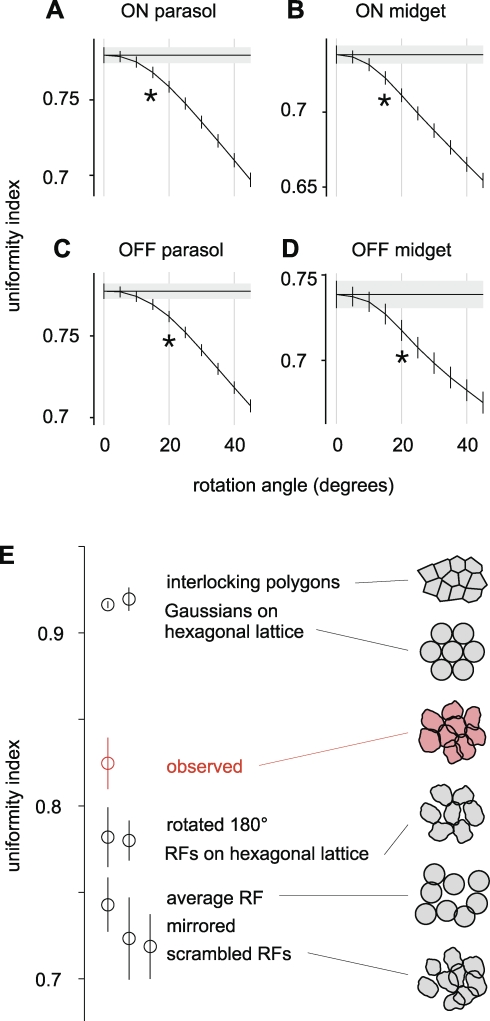
Precision and Theoretical Bounds on RF Coordination (A) Test to identify the minimum perturbation that significantly disrupts the uniformity of coverage. For on parasol preparations, the UI is plotted as a function of the angle by which RFs were rotated around their center points. Data from clockwise and counterclockwise rotations were pooled (see Materials and Methods). An angle as small as 15° significantly reduced the UI (*p* <0.01); this angle is represented as an asterisk (*) beneath the curve. Horizontal black line and gray rectangle indicate the mean and error bars on the unrotated condition. (B–D) Same analysis as in Figure 4A applied to other cell types. (E) UI values of observed RFs and various simulated RFs from a representative population of on parasol cells. For a meaningful comparison to the data, noise was added to simulated RFs to match the noise in the observed RFs (see Materials and Methods). As a result, even the most regular arrangement (“interlocking polygons”) produced UI values lower than 1. Error bars represent the SEM within each population (see Materials and Methods).

Examination of the entire population, however, revealed an elegant resolution to the problem of irregular RF shapes: RFs were coordinated, interlocking to sample visual space more uniformly (Figures 1F and 2). To visualize this coordination, each RF was summarized by interpolating a contour line at a single sensitivity level, similar to an iso-elevation line on a topographical map. For each cell type, a single contour level was selected that, on average, assigned each spatial location to a single cell. The contour lines for all RFs of a single type were then plotted together to illustrate the structure of the collective visual sampling. As expected from previous anatomical and physiological studies, the locations of the RFs of each cell type formed an approximately regularly spaced lattice (Figures 1F and 2) [7,11,13,14]. Surprisingly, however, RFs showed a striking tendency toward coordinated structure: irregular outlines of neighboring cells complemented each other, interlocking like jigsaw puzzle pieces. RFs were precisely coordinated in all four major cell types (Figure 2). There appeared to be no coordination between cells of different types, emphasizing the importance of clearly distinguishing one cell type from another when studying sensory encoding by a neural population.

The observed coordination of RFs produced more uniform visual sampling than expected by chance, as demonstrated using a geometric test. The null hypothesis was that visual sampling is no more uniform than expected from random interaction between irregular RF shapes, where “irregular” is defined as deviation from circular symmetry. Under this null hypothesis, mirroring each RF around its center point should not affect sampling uniformity [6]. To test this hypothesis, the arrangement of simultaneously recorded RFs of a single type (Figure 3A, first column) was compared to the arrangement obtained after each RF was artificially mirrored (Figure 3A, second column). Visual inspection showed that mirroring severely disrupted visual coverage: the area covered by exactly one RF contour (gray) was significantly reduced, and there were many more gaps (black) and overlaps (white). Thus RF shapes were not arranged randomly, but rather were coordinated in a way that provides more uniform coverage of visual space.

The spatial features of RFs that are important for uniform visual coverage were not captured by the most common and accurate idealized RF model, an elliptical difference of Gaussians [5,14,17]. This was demonstrated using a second test in which the null hypothesis is that deviations from elliptical (rather than circular) symmetry do not produce more uniform coverage. The hypothesis was tested by rotating each RF by 180° around its center point, a perturbation that leaves elliptical shapes intact but disrupts any coordination between the nonelliptical structure of adjacent cells. Rotating each RF substantially disrupted coverage (Figure 3A, first and third columns), rejecting the null hypothesis. Thus, although RF shapes exhibited apparently “noisy” deviations from smooth elliptical models, these deviations were coordinated to produce a more uniform sampling of visual space (see Figure 4, and below).

The above observations were confirmed quantitatively using a numerical measure of the regularity of visual coverage: the uniformity index (UI) (Figure 3B). For a collection of RFs represented by a single contour level, the UI is the proportion of visual space covered by exactly one contour, computed only in regions where all cells of a type were apparently recorded (see Materials and Methods). Graphically, the UI represents the fraction of space in Figure 3A that is colored gray. Higher UI values indicate more uniform coverage; if RF shapes interlocked perfectly, the UI would equal 1. Scatter plots in Figure 3B show that the UI was always reduced when RFs were mirrored or rotated by 180°, confirming the visual inspection of perturbed RFs. This finding was not affected by setting the threshold level defining RF contours substantially above or below its optimal value (see Materials and Methods).

The striking coordination of RF structure suggested that retinal circuits may sample the visual scene with high precision, perhaps in a manner that approaches the optimum for high-resolution vision. To measure the precision of interlocking, RFs were artificially perturbed, and the minimal perturbation that significantly disrupted visual coverage was identified (Figure 4A–4D). Rotating RF shapes around their respective center points by as little as 15° led to a significant reduction in the UI. The minimal angle was similar for both parasol and midget cells, showing that in both populations, the arrangement of RF shapes is exquisitely tuned to sample visual space more uniformly.

Cell to cell variability contributed importantly to uniform coverage. When each RF in the observed population was replaced with the average RF, the UI was substantially reduced (Figure 4E, “observed” vs. “average RF”). This observation suggests that uniform sampling is more important for visual encoding than homogeneous RF shapes.

The observed RFs approached an optimal arrangement. This was demonstrated by comparing the UIs of various simulated populations. RFs are commonly modeled as a hexagonal lattice of identical circular difference of Gaussians functions. Because of regular spacing and regular shapes, this idealization produces a very uniform sampling (Figure 4E, “Gaussians on hexagonal lattice”). When the smooth ideal RFs were replaced with the observed irregular and variable RFs, uniformity dropped substantially (Figure 4E, “RFs on hexagonal lattice”). When these RFs were placed on the observed quasiregular lattice, uniformity fell further (Figure 4E, “scrambled RFs”). Thus, as expected, uncoordinated irregular RF shapes can degrade the uniformity of visual sampling [3]. With coordination, however, uniformity increased substantially. Compared to the baseline of uncoordinated RFs (Figure 4E, “scrambled RFs”), the observed RFs exhibited uniformity 53% of the optimum given by perfectly interlocking shapes (Figure 4E, “interlocking polygons”). Thus, the coordination of retinal RFs produces a substantially more uniform visual representation than would occur if RFs were independently formed.

## Discussion

The present results demonstrate that the visual representation in the primate retina is finely coordinated to achieve a homogeneous sampling of visual space. This finding has several important implications for retinal circuitry, retinal development, and the precision of neural population codes.

The discovery of RF coordination is distinct from previous studies of RF overlap. Those studies focused on the *average* degree of overlap between neighboring RFs within a population [15,18–21]. Complementing the empirical measurements, several studies have suggested that the observed average overlap may be nearly optimal (e.g., [18,21–23]). For example, it has been suggested that the observed spacing of RGC RFs can produce a relatively uniform sensory surface [18], without excessive spatial pooling [21], and may maximize the information transmitted from the retina to the brain about natural scenes [21]. It should be noted that the present recordings exhibited RF overlap consistent with previous studies (e.g., [15]). However, the overlap was not apparent in the figures because the contour visualization focused on RF shape alone. Overlapping RFs may be important for representing fine spatial detail [21], but RF overlap was not the primary focus of the present study. Instead, the present study focused on the coordinated fine structure of *individual* RFs relative to their neighbors, a property that is independent of the average overlap. The coordination of RF shapes produces more consistent sampling of visual space, contributing to the uniformity of the visual representation irrespective of the average overlap. Although there is undoubtedly a link between this uniformity and the neural representation of visual space, new theoretical frameworks will need to be developed to assess exactly how RF coordination improves visual encoding.

What retinal mechanisms precisely coordinate RF shapes? One study comparing the RF and DF shapes of individual rabbit ganglion cells suggested that DFs do not determine the fine structure of RFs [3]. However, it is possible that primate parasol and midget cells exhibit a different relationship between RF and DF. In fact, the interlocking midget cell RFs observed here exhibited deformations similar in size and shape to the interlocking midget cell DFs observed previously [7]. This suggests that, at least among midget cells, RF shapes might match DF shapes at a fine scale. Although parasol cell DFs have too much overlap to interlock in this fashion [11], interactions among dendrites of neighboring parasol cells could contribute to complementary RF shapes. Thus it will be interesting for future studies to determine how the relationship between RF and DF varies among different cell types, particularly those in which RFs interlock with neighbors.

Alternatively, the precise coordination of RF shapes could rely primarily on the layers of circuitry that connect photoreceptors to ganglion cells [24]. For example, the regular lattice of bipolar cells [25–27] might contact ganglion cells in a partly exclusive fashion, so that two neighboring ganglion cells would not both receive strong input from the same bipolar cell [27,29]. This hypothesis is supported by the finding that the spatial arrangement of bipolar cell synapses onto ganglion cells is highly variable [30,31], consistent with each ganglion cell requiring a unique pattern of bipolar cell inputs to achieve coordination with its neighbors. Another possibility is that inhibitory amacrine cell inputs reduce the sensitivity of RF lobes that would otherwise jut too far into a neighboring RF [32]. In any case, the discovery of RF coordination provides a guiding framework for understanding the retinal circuit elements that determine the fine features of RF shape.

How might RF coordination arise during development? Given the diversity of circuit elements that must be arranged to precisely align interlocking RF shapes, one possibility is that RF shapes arise from plasticity driven by visual input [33,34]. Under this hypothesis, the mechanisms that modify retinal circuitry would be sensitive to the coordination of visual signals in neighboring RFs, as distinct from anatomical growth cues or patterns of spontaneous activity [35]. It may be interesting to test this hypothesis by investigating how early in development RF shapes appear to be coordinated and how the coordination is affected by rearing in light- or form-deprived visual environments [36].

The present results have surprising implications for how populations of neurons produce an efficient and complete representation. Recorded in isolation, single neurons frequently exhibit irregular response properties, suggesting that large populations must rely on averaging or interpolation to produce accurate sensory performance or behavior (e.g., see [37–39]). The present results, however, show that in a complete population, irregular features can be integral to a finely coordinated population code. This suggests that the nervous system operates with a higher degree of precision than previously thought, and that irregularities in individual cells may actually reflect an unappreciated aspect of neural population codes (e.g., [40]).

## Materials and Methods

### Ethics statement.

In accordance with institutional guidelines, retinas were obtained from deeply anesthetized monkeys (Macaca mulatta, Macaca fascicularis) being euthanized for other experimental procedures, as described previously [15].

### Summary.

Eyes were enucleated and hemisected, and the vitreous was removed in room light. Retinas remained attached to the pigment epithelium and were incubated in the dark for at least 30 min prior to recording. For recording, patches of peripheral retina 3–5 mm in diameter and 6–12 mm from the fovea were isolated from the pigment epithelium and held flat against a planar array of 512 extracellular recording electrodes. The preparation was perfused with oxygenated and bicarbonate-buffered AMES medium (Sigma; [pH 7.4] 32–35 °C). The visual stimulus was produced using the optically reduced image of a computer display focused on the photoreceptors. Voltage data for each electrode were digitized at 20 kHz. Offline, spike waveforms were sorted into clusters in a multistep procedure [15], and clusters with a minimum refractory period between spikes were identified as single neurons. All analyses were performed using custom software.

RFs were mapped by computing the spike-triggered average (STA) stimulus obtained in the presence of a white noise stimulus. Features of the STA were parameterized using a separable model consisting of a two-dimensional difference of Gaussians spatial profile, a biphasic time course, and a spectral profile. For analysis of RF shape, the spatial component of the STA was extracted using singular value decomposition (SVD) across time (see below). Light sensitivity was normalized across neurons by regressing the RF against an elliptical single Gaussian fit with a peak of 1. RFs were low-pass filtered by convolving with a two-dimensional Gaussian function. The standard deviation (SD) of the Gaussian was typically 0.3 to 0.9 pixels. After spatial smoothing, contour lines were linearly interpolated in each RF. For each cell type examined, a common contour level was applied to all cells and adjusted to maximize the UI. The UI was equal to the proportion of space covered by exactly one contour (excluding both gaps and overlaps). It was computed only within the area in which all cells appeared to have been recorded, as defined by an automated procedure (see below). The contour level that maximized the UI tended to produce RF contours that just touched their neighbors, providing the greatest amount of information about whether RF shapes were complementary. If a contour level significantly higher or lower had been used, the area covered by exactly one cell would have been very small, yielding little or no information about the coordination of RF shapes. For analysis of mirrored or rotated RFs, the contour level used for the UI calculation was reoptimized after transforming the RFs.

Simulated RFs (Figure 4E) were defined in spatial bins the same size as the pixels of the measured RFs, and independent Gaussian noise was added to each bin to match the noise in measured RFs. For reshuffling of measured RFs, each RF was translated to its new location without additional noise. The UI of simulated RFs was computed using the same procedure as for the measured RFs. In particular, the contour level used for the UI calculation was reoptimized after RFs were altered.

### RF characterization.

Within each pixel of the flickering checkerboard stimulus, the red, green, and blue monitor primaries were modulated based on random draws from a binary distribution, chosen independently in space and time. Pixel sidelengths ranged from 18 to 60 μm (on the retina) and the color changed every 8.33 to 50 ms. For each neuron, the spatiotemporal RF was estimated by computing the STA stimulus over the 250 ms preceding a spike. This RF included the contribution of both center and surround. Although the surround time course was delayed compared to the center time course, the stimulus update temporal period was sufficiently long that this delay typically did not significantly influence the spatial RF estimate.

Some RFs analyzed here were originally recorded for use in other studies, but could also be used to study RF shape coordination. A recording was used only if it satisfied two criteria: (1) the pixels were small enough to resolve the fine shape of each RF (generally at least four to five pixels per RF diameter), and (2) the measurements of RFs exhibited low noise. These criteria were met by stimuli of various spatial and temporal scales, producing a large range of stimulus parameters in the dataset.

The color value of each pixel was chosen from a binary distribution rather than a Gaussian distribution to more rapidly characterize RF shape. The small pixel sizes used produced a relatively low effective contrast, and thus responses were approximately linear. In the linear regime, a binary distribution produces an unbiased estimate of RF shape [41].

Each space–time STA described both the RF shape and kinetics. To extract only the spatial component, the STA was approximated by a space–time separable function. First, the STA was put into a matrix *M* in which each row was the time course of a single pixel. The singular value decomposition (SVD) was performed, yielding a standard decomposition *M* = *UDV^t^*, where *U* and *V* are orthogonal matrices and *D* is diagonal. The first column of *U* contained the primary spatial component of the STA. Visual inspection showed that this spatial component resembled individual frames of the STA movie in which the spatial structure of the RF was most clear.

### The uniformity index.

The UI was computed for the mosaic formed by each cell type in two steps. The first step identified regions in which all cells appeared to have been recorded. The second step revealed the contour level that maximized the area covered by exactly one cell. The details of these steps and justifications are described below.

In some preparations, a complete lattice of RFs was recorded (e.g., Figure 2A), whereas in other preparations, many RFs appeared to be missing (e.g., Figure 2D). This variability was likely due to mechanical factors, such as contact with the electrode array. A lattice of RFs was only included in the analysis if sufficiently many RFs appeared to have been recorded, usually about 50%; and in each preparation, only regions with contiguous RFs were used for quantitative analysis. If RFs were precisely uniformly spaced, contiguous regions would be easy to identify. However, RFs exhibited somewhat variable spacing, and thus a multi-step algorithm was required to exclude regions of space not covered by recorded cells.

First, the entire recorded region was subdivided into triangular areas using the Delaunay triangulation [15,42] of the collection of Gaussian fit center points. The area within a triangle was considered to be covered by contiguous RFs only if the cells at its vertices were sufficiently close together, with the cutoff distance equal to 1.9 times the median nearest neighbor spacing. The parameters of this algorithm were chosen based on the statistics of RF mosaics, as described below.

The median was used to estimate the typical nearest-neighbor spacing because it is relatively robust to outlying points. In a complete mosaic, nearest-neighbor distances follow an approximately Gaussian distribution. In an incomplete mosaic, however, nearest-neighbor distances will form a distribution composed of a Gaussian plus a long tail that corresponds to cells whose nearest neighbors were not recorded. Thus the median was the most robust estimate of nearest-neighbor spacing.

**Figure 5:**
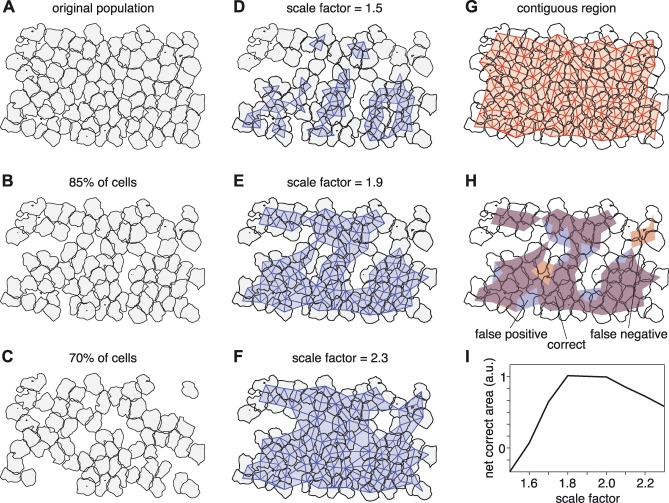
Algorithm for Selecting Regions of Contiguous Cells (A) A complete collection of simultaneously recorded on parasol cells (also shown in Figure 2A). (B and C) The same population, randomly subsampled to 85% and 70%, respectively. (D–F) Estimates of the region of contiguous cells from the population in (B), as determined using an automated algorithm with scale factors of 1.5, 1.7, and 1.9 (see text). (G) The true region of contiguous cells for the population in (A). (H) Comparison of the true region of contiguous cells for the population (shown in [B]) and the result of the algorithm using a scale factor of 1.9 (shown in [E]). In purple areas, the algorithm performed correctly; in red areas, the algorithm missed contiguous cells; and in blue areas, the algorithm identified noncontiguous cells as contiguous. (I) Quantification of the algorithm's performance for a range of scale factors. The abscissa shows the scale factor, the ordinate shows the area of correctly identified contiguous cells minus the area of false negatives and false positives (see text). The area is in arbitrary units (a.u.).

The scale factor, 1.9, was chosen empirically to match the observed variability of cell spacing. Figure 5 demonstrates the procedure that was used to arrive at this number. To simulate an observed mosaic with missing cells, a complete mosaic (Figure 5A) was randomly subsampled. For each subsampled mosaic, a range of scale factors was used in the algorithm to identify regions of contiguous cells. Figure 5D–5F shows an 85% subsampled mosaic in which scale factors of 1.5, 1.9, and 2.3 were used, respectively. The areas identified as containing contiguous cells are colored in blue. For a low scale factor (Figure 5D), the colored region is clearly too small; several groups of contiguous cells were missed. For a high scale factor (Figure 5F), the colored region is too large, and includes several gaps where cells were not recorded. An intermediate scale factor of 1.9 appears to describe the region of contiguous cells most accurately (Figure 5E).

Quantifying this trend revealed that a scale factor of 1.9 was optimal. For each subsampled mosaic, the region of truly contiguous cells was identified by testing which cells were missing from the original, complete mosaic. As an example, Figure 5G shows the region of truly contiguous cells for the mosaic shown in Figure 5A. For the subsampled mosaic shown in Figure 5D–5F, the region of truly contiguous cells is shown in Figure 5H in red and purple. For each scale factor, the estimated region of contiguous cells was compared to the actual region, thus creating three areas: the area correctly identified as containing contiguous cells, the area of false negatives, and the area of false positives. Figure 5H shows these three areas for the scale factor value 1.9. The relative sizes of the three areas were summarized by taking the correct area minus the sum of the error areas. Figure 5I shows this summary value for several scale factors, ranging from 1.5 to 2.3. Data were pooled from mosaics subsampled at 100%, 85%, 70%, and 55%. Higher numbers represent more accurate identification of contiguous regions, and the curve shows that scale factors of 1.8, 1.9, and 2.0 are approximately equally effective. Thus a value of 1.9 was chosen to robustly identify regions of contiguous cells.

Only regions of contiguous cells were used for subsequent analysis. The degree of RF interlocking, and thus the uniformity of RF coverage, was measured by considering how precisely RF shapes fit together. To efficiently describe the RF shapes, each RF was represented by a single contour level at which neighboring cells just touched. To avoid bias, this was done automatically by finding a single contour level for all cells that maximized the total area covered by exactly one cell.

**Figure 6:**
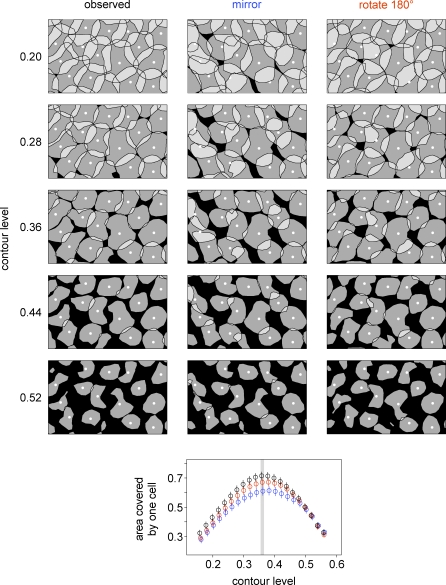
Analysis of RF Coordination Was Insensitive to the Contour Level Top panels show a population of on parasol cells (12-mm eccentricity) in which each RF is drawn at the contour level indicated by the left-side labels. The first column shows observed RFs, and the second and third columns show RFs mirrored or rotated 180°, respectively. The width of each panel represents 660 μm on the retina. The bottom scatter plot shows how the area covered by exactly one cell varied with different contour levels, for both the observed and perturbed RFs. The contour level that maximized the area covered by one cell, 0.36, is indicated with a gray line. At each contour level, the area covered by one cell is plotted for observed RFs (black), mirrored RFs (blue), and rotated RFs (red). Error bars represent the SEM within each population (see Materials and Methods). Perturbing RFs reduced the area covered by a single cell over a wide range of contour levels surrounding the optimum.

To visualize why this was the most informative contour level, Figure 6 shows a collection of on parasol RFs at a variety of contour levels. For low contours (left column, upper rows), each RF contour was large, and the contours overlapped so much that RF shape interactions were difficult to distinguish. For high contours (left column, lower rows), each RF was too small for neighbor relationships to be revealed. The contour level that provided the most information about RF shape interlocking was the level at which RFs on average just touched their neighbors (0.36, left column, center row), and equivalently, the level that maximized the area covered by exactly one cell (scatter plot).

The optimal contour level was different for each preparation and varied from 0.16 to 0.40 (median 0.24). Note that the absolute contour value reflects several factors, including the amount of noise in the measurement (and thus the duration of the recording), the degree of blurring that was applied, and the degree of RF overlap.

The observation of RF coordination was not sensitive to the particular contour level chosen, as illustrated in Figure 6. Over a broad range of contour levels, the area covered by one cell always declined when RFs were mirrored or rotated (bottom plot). At extreme contour levels, however, there was no effect of mirroring or rotation, because very high or low contours do not reveal the interlocking of RF shapes (upper and lower rows).

Error bars for the measured UI were produced as follows. Within each Delaunay triangle, a local UI was computed as the area covered by exactly one cell within the triangle. The overall reported UI was computed across the area occupied by all triangles. Error bars were equal to the standard error of the mean (SEM) of a subset of the local UIs. The subset was chosen so that no two triangles shared an edge, ensuring that local correlations in the UI did not artificially reduce the SEM. The SEM was validated using a bootstrap simulation. The simulation concluded that the SEM was a conservative estimate, typically overestimating the standard deviation of the UI by 30% to 60%.

### Rotation plot (Figure 4A–4D).

Each RF was rotated about its Gaussian fit center point by the same angle; and for each such rotation, the contour level was chosen to maximize the UI. After generating rotated contours, the following procedure was applied to each cell type. First, data were pooled from all preparations in which sufficiently many cells of that type were recorded. A subset of the Delaunay triangles was chosen in each preparation as described above, and the local UI values of these triangles were averaged from all datasets to determine the mean UI at each rotation angle (including 0°), with error bars equal to the SEM. For each nonzero rotation angle, a one-tailed two-sample *t*-test was used to determine whether the UI was significantly lower (*p* < 0.01) than the UI of the unrotated RFs.

### Generation of simulated RFs.

*Mirror test.* Each RF was mirrored about an axis passing through the Gaussian fit center point. The angle of the mirror axis was chosen using a procedure that ensured it was both arbitrary and unique. For visualization (Figure 3A), the axis was parallel to the short edge of the boundary of the region shown. For quantitative analysis (Figure 3B), the axis was parallel to the short edge of the recording array.

*Interlocking polygons.* Voronoi domains were computed based on the center points of measured RFs. Each RF was shaped like the Voronoi domain with amplitude following a Gaussian taper matched to the taper of the observed RFs.

*Gaussians on a hexagonal lattice.* Lattice spacing was equal to the median nearest-neighbor spacing of the measured RFs. Each RF was a circular difference of Gaussians function with center radius equal to 0.5 times the lattice spacing, surround radius equal to twice the center radius, and surround amplitude equal to 0.2 times the center amplitude.

*RFs on a hexagonal lattice.* Cell centers were located on a regular hexagonal lattice, and the cell at each location was a randomly chosen RF from the original population.

*Average RF.* Each RF was replaced by the average RF with noise added to match the noise in observed RFs.

*Scrambled RFs.* Each RF was replaced by a randomly chosen RF from the same preparation. The lattice of RF center locations was held constant.

## Abbreviations

DF: dendritic field
RF: receptive field
SEM: standard error of the mean
STA: spike-triggered average
UI: uniformity index
